# Supplementary material for: Ultrafast Photo-Ion Probing of the Relaxation Dynamics in 2-Thiouracil
Source: Molecules. 2023 Mar 3;28(5):2354. doi: 10.3390/molecules28052354 (PMC10005304; doi:10.3390/molecules28052354)
Supplement: Supplementary file 1 [file molecules-28-02354-s001.zip › molecules-2111935-supplementary.pdf]

Ultrafast photo-ion probing of the relaxation dynamics in 2-thiouracil: Supplementary Data

Here we present additional data and commentary from the UV/UV pump-probe ion mass spectroscopy experiments, to complement and expand the main article.

1. Power Series

The power series plots seen in Figures S1-S6 represent the full data set discussed in Subsection 2.3 of the main article, showing how the *Signal* for each ion of interest varies with respect to the combined pump-probe laser energy. The data is displayed in a "log-log" format and each figure is divided into six sections, representing data collected in the power series at different pump-probe delays, including a) Time zero, b) 100 fs, c) 500 fs, d) 1 ps, e) 5 ps and f) 10 ps. Main data points are shown as blue dots and is above background noise and below saturation. A linear fit of these data points is represented by the solid red line, with the value of the fitted gradient, *m*, being shown in the bottom right corner of each plot (the same value seen in Table 2 of the main article).

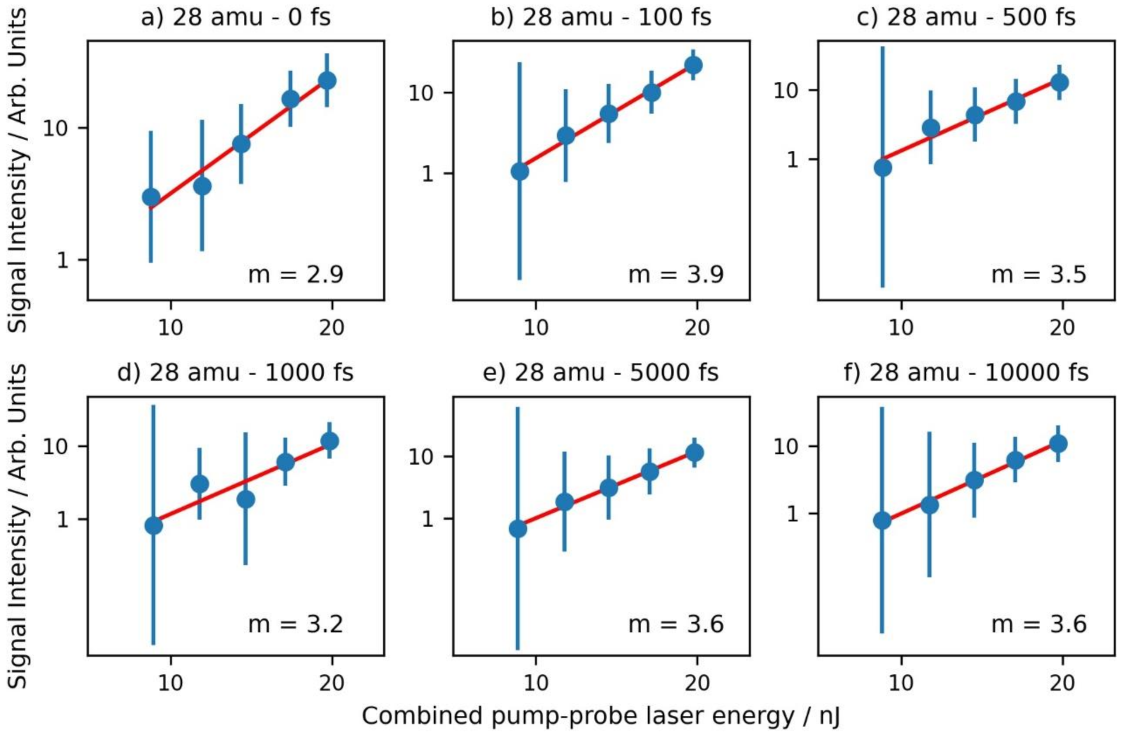

Figure S1: Power series for the 28 amu fragment ion.

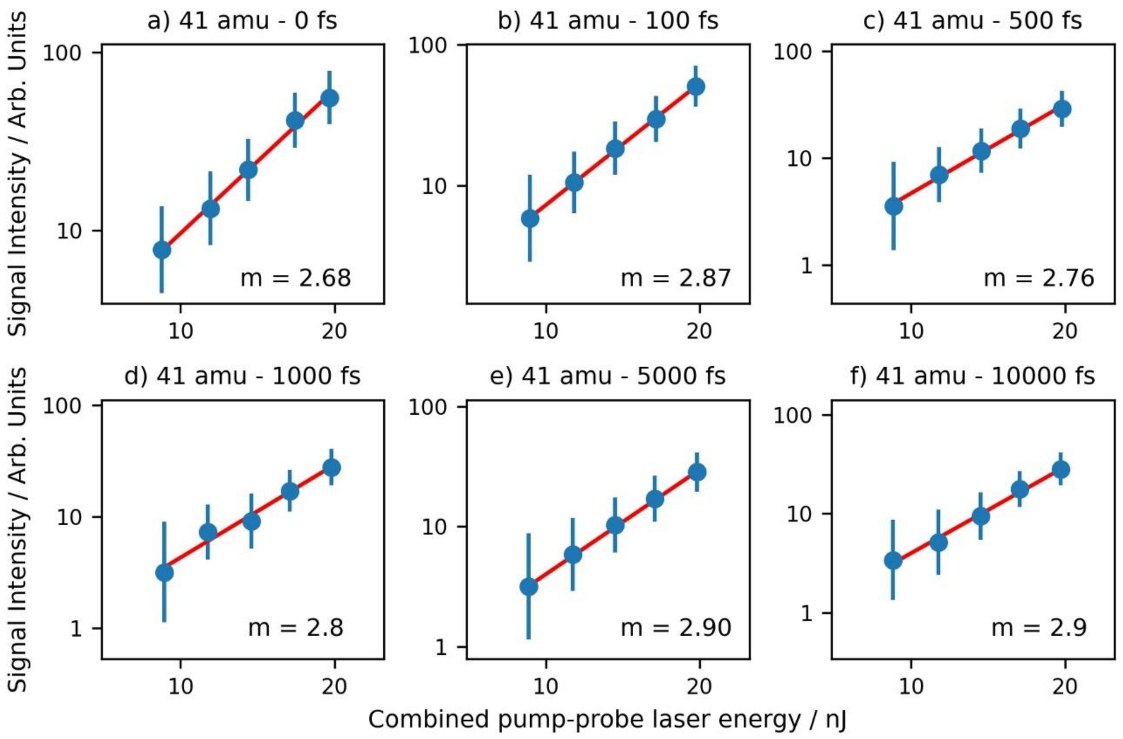

Figure S2: Power series for the 41 amu fragment ion.

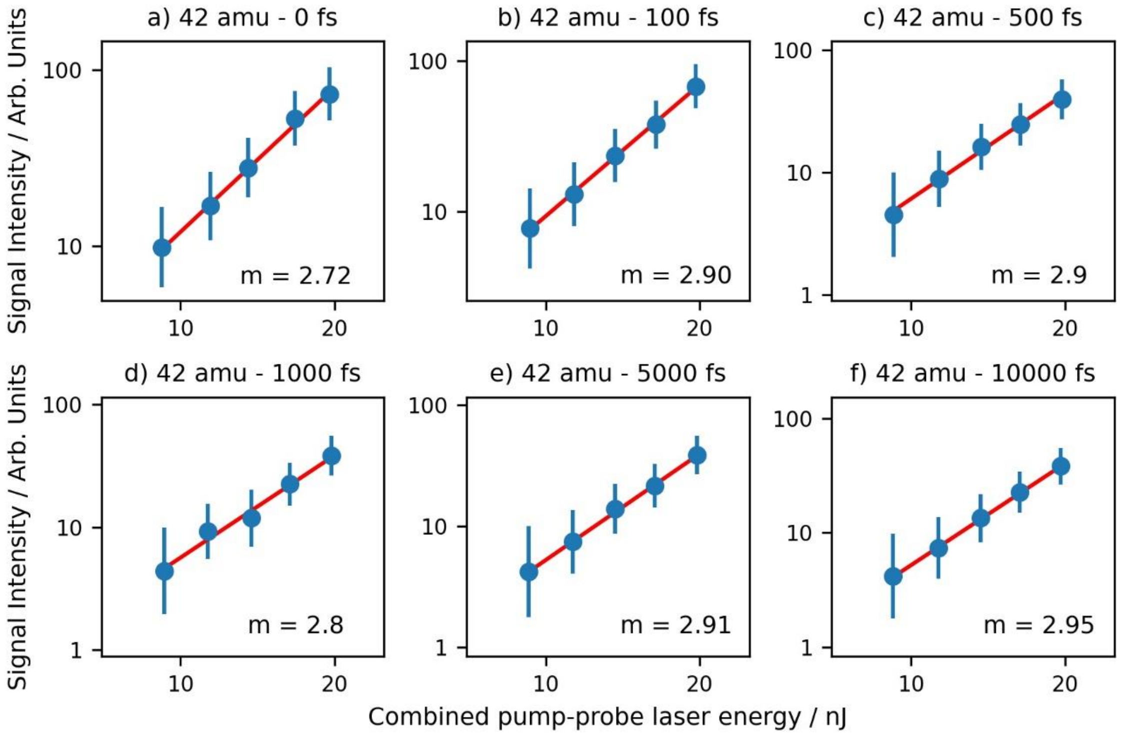

Figure S3: Power series for the 42 amu fragment ion.

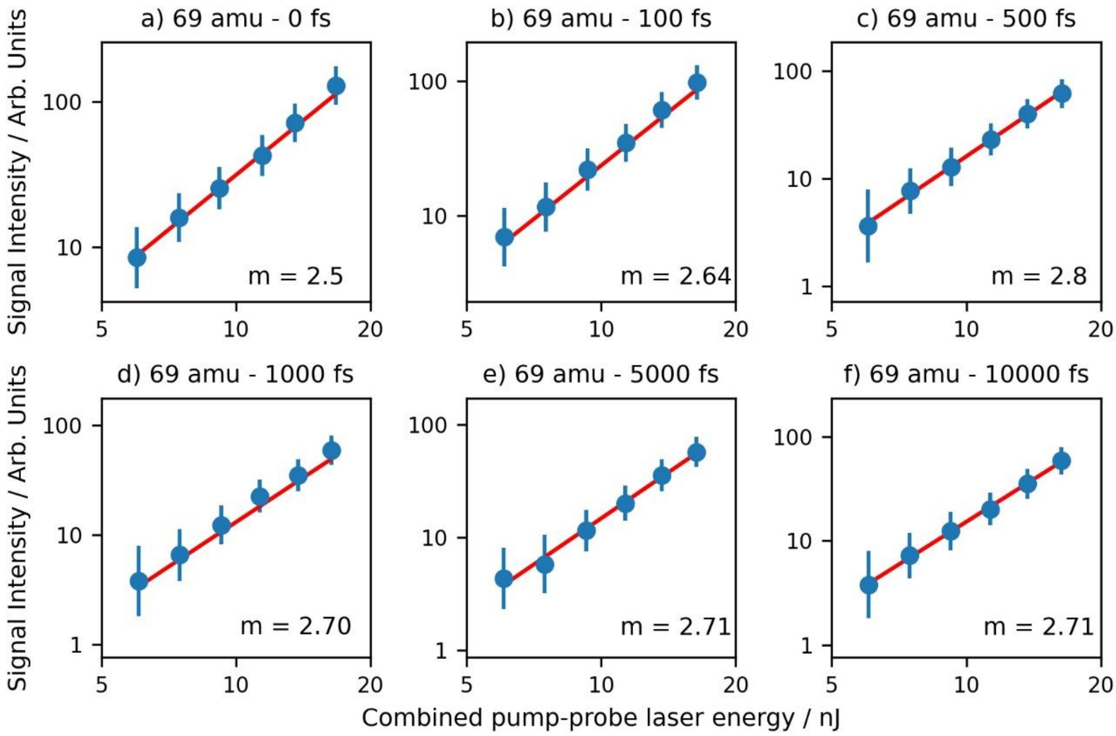

Figure S4: Power series for the 69 amu fragment ion.

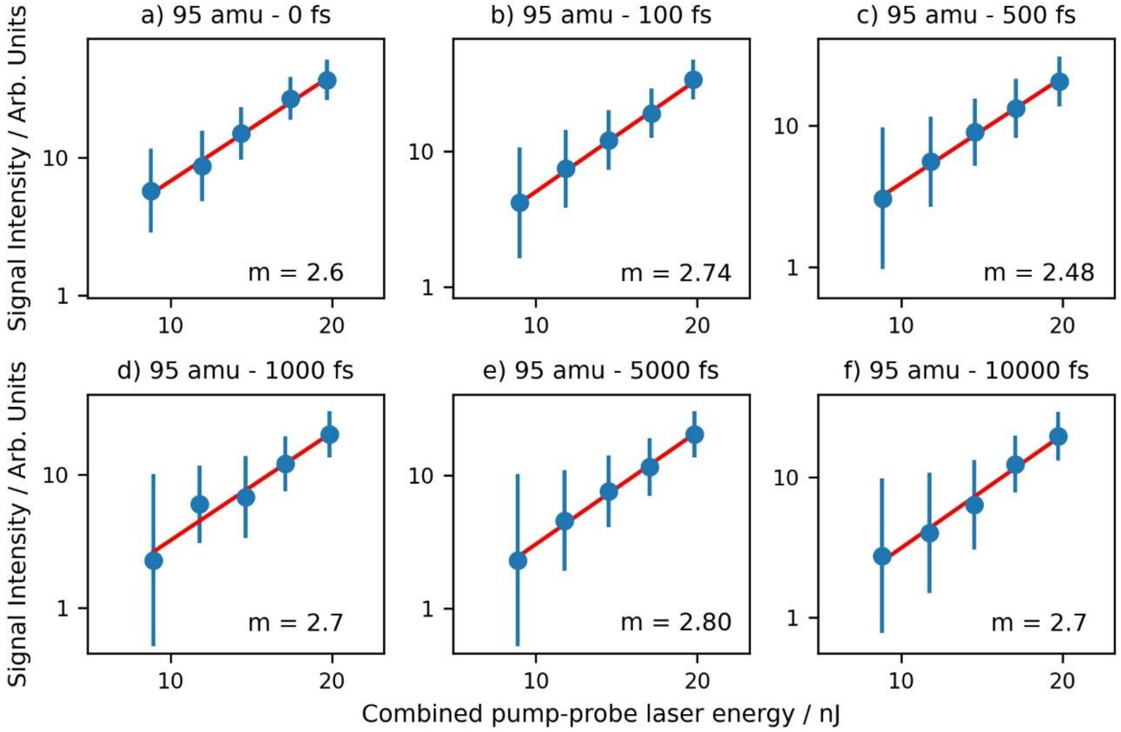

Figure S5: Power series for the 95 amu fragment ion.

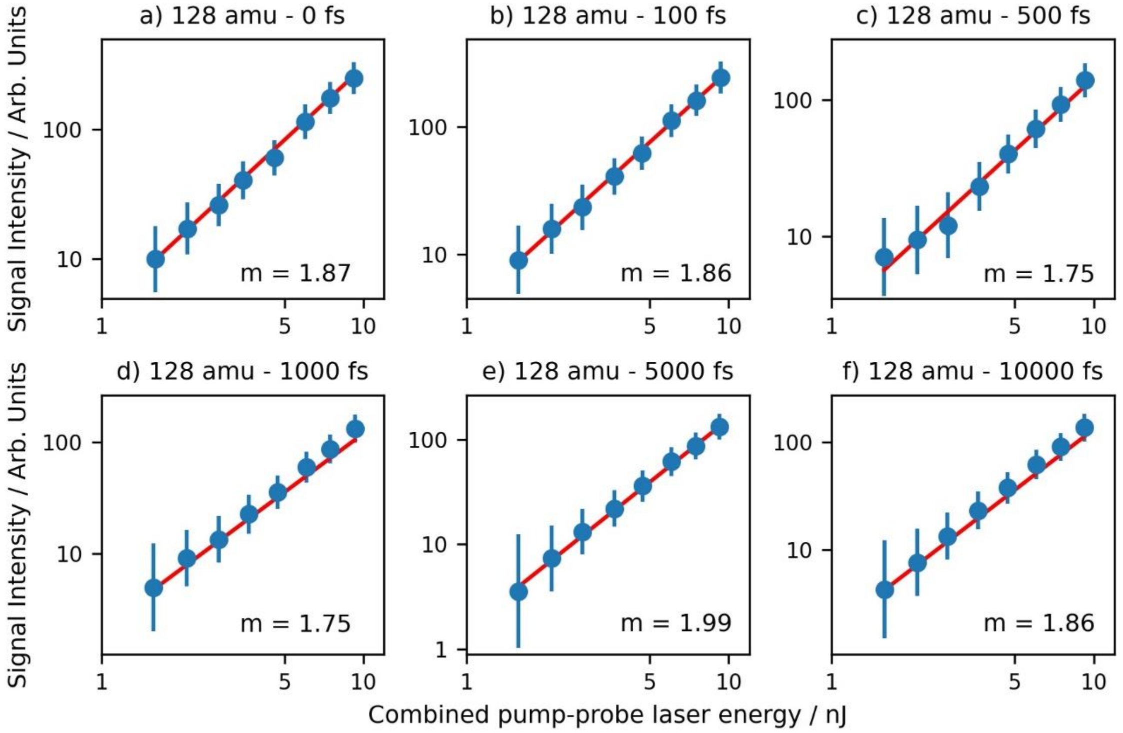

Figure S6: Power series for the parent (128 amu) ion.

2. Time-dependent UV/UV signal from pump-probe beams with equal power

The following relates to results discussed in Subsection 2.4. of the main paper. In Figures S7-S9, we present the remaining time-resolved signals for the 41, 42 and 95 amu fragment ions. The original data is represented by the blue dots, the fit of this data by the red line, and the background signal by the blue line. Inserts show the respective signal for the fragment ions extending out to 100 ps. It should be noted that for the 95 amu (Figure S9), a dip in the signal below the background level in the main plot between -0.75 and 1.5 ps is observed, but not for the longer scan seen in the insert performed between 0 and 100 ps. Despite both scans using the same laser power, this dip is likely caused by a saturation of the weak 95 amu signal, possibly due to slight focusing differences caused by laser-alignment checks performed between the two scans. Whilst unfortunate, we do not believe that this saturation affects the results significantly, as the decays observed for the 95 amu is similar to the other fragments.

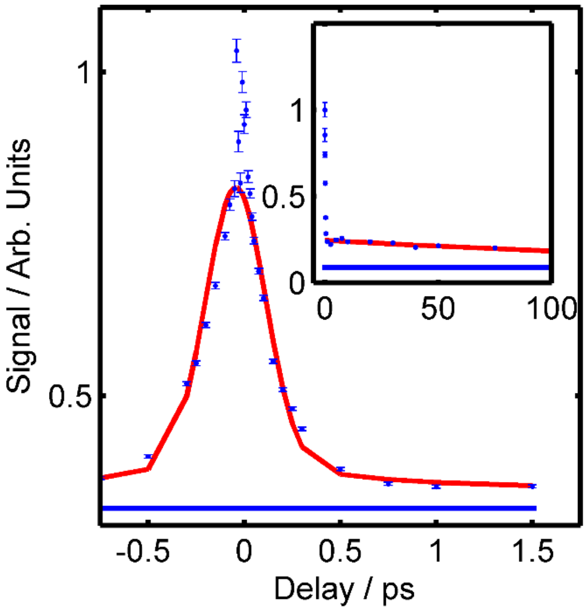

Figure S7: Time-dependent decay of the 41 amu fragment ion.

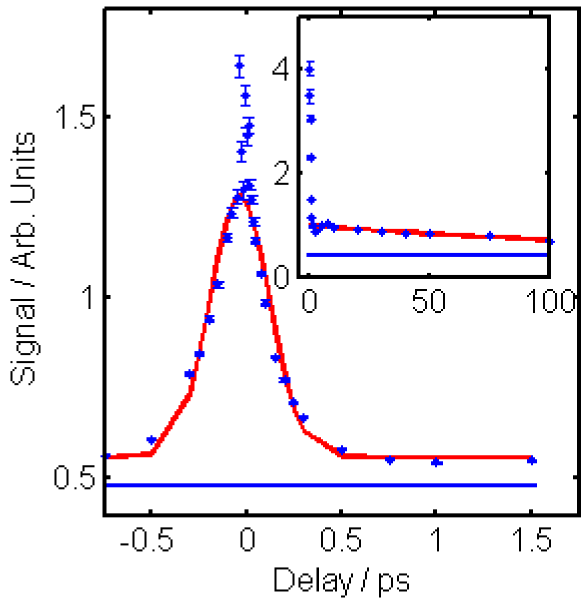

Figure S8: Time-dependent decay of the 42 amu fragment ion.

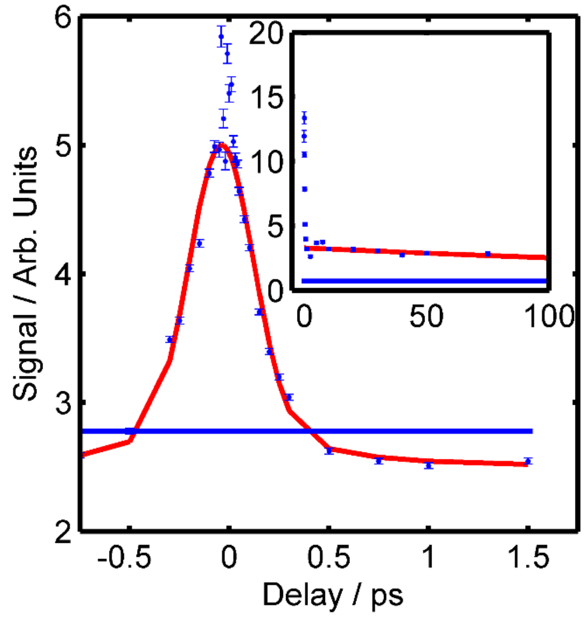

Figure S9: Time-dependent decay of the 95 amu fragment ion.

3. Time-dependent UV/UV signals from pump and probe beam with unequal powers

The following relates to results discussed in Subsection 2.5. of the main paper. In Figures S10-S13 we present the time-dependent signals observed for the 28, 41, 42 and 95 amu fragment ions when unequal pump and probe powers are used. In the images below, 3 nJ was used in the probe pulse, whilst 6 and 9 nJ per pulse was used in the pump beam. Experiments with 1.5 and 3 nJ per pulse in the pump beam were also performed, however, no signal above the background was observed for these fragments, and hence not presented. In each image, two plots are shown which dictate whether the pump pulse arrives first (pump-probe, blue) or if the probe pulse arrives first (probe-pump, orange).

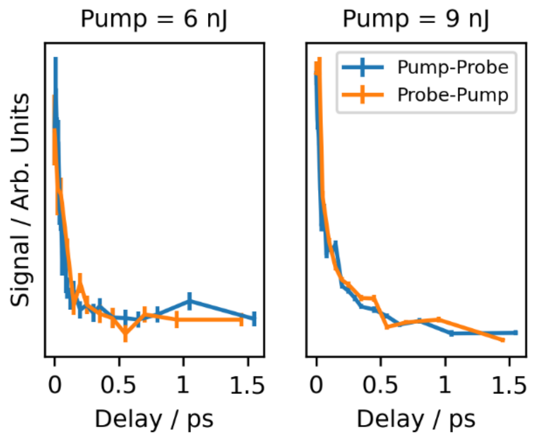

Figure S10: Inhomogeneous pump-probe signal for the 28 amu fragment ion.

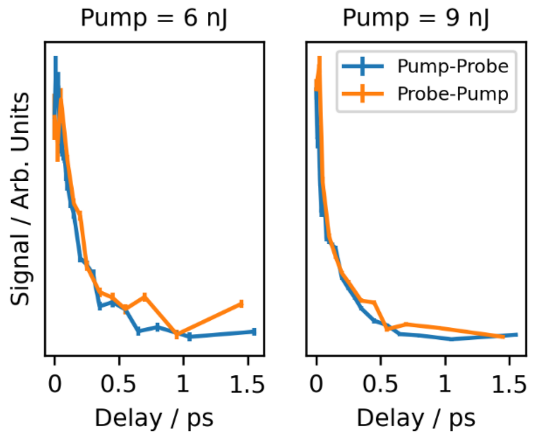

Figure S11: Inhomogeneous pump-probe signal for the 41 amu fragment ion.

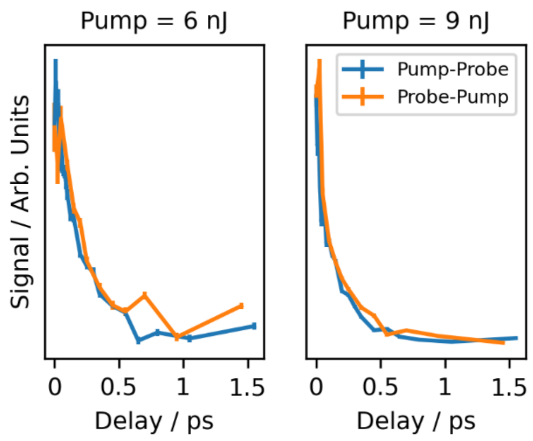

Figure S12: Inhomogeneous pump-probe signal for the 42 amu fragment ion.

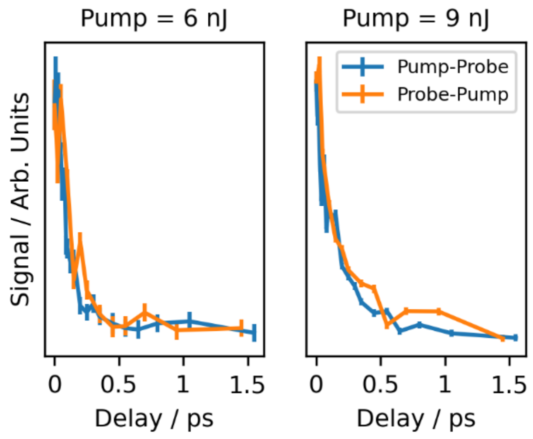

Figure S13: Inhomogeneous pump-probe signal for the 95 amu fragment ion

4. Novelties of the 100 amu fragment

Due to its weak pump-probe signal, we did not explicitly discuss the 100 amu fragment ion in the main article. However, its appearance in the first place, especially in comparison to studies on uracil, is interesting, sparking questions as to how the fragment ion is formed, and what chemical formula can be attributed to it.

To produce the 100 amu fragment ion a 28 amu fragment must be lost from the parent 2-TU molecule. This is the first point of interest. When making comparisons to uracil, a loss of 28 amu from uracil would result in an 84 amu fragment ion. However, to our knowledge, the 84 amu fragment ion from uracil has not been observed in any single-photon ionisation or particle impact study [1–6]. This is in stark contrast to the results presented in Subsection 2.2., where the 100 amu fragment ion of 2-TU appears in the single-photon VUV experiments.

Whilst not observable in single-photon experiments, Eden *et al*. have demonstrated that the 84 amu fragment ion of uracil can be observed in select multi-photon ionization experiments using nanosecond pulses at a wavelength of ≤232 nm (i.e. ≥5.34 eV) [7–9]. Femtosecond pump-probe studies with a delay of up to 100 ps between the two pulses failed to produce the fragment, leaving them to suggest that the fragment is created through a long-lived excited state in uracil after dynamics are initially launched on the S_3 rather than S_2 state [8]. With the use of high-resolution mass spectra of both uracil and deuterated

uracil, Eden *et al*. were able to attribute the 84 amu fragment ion to $C_3H_4N_2O^+$, ultimately suggesting that the loss of the 28 amu was due to a CO ejection after a ring-opening process involving through the N_3-C_4 bond [7–9].

Due to the same mass loss, it is possible that the 100 amu fragment ion of 2-TU is also created through the ejection of CO, leaving behind $C_3H_4N_2S$, which is then ionized by the probe pulse. It is also possible, due its appearance in the multi-photon UV/UV experiments, the 100 amu fragment ion of 2-TU is created through a similar type of ring-opening process that Eden *et al*. suggested for uracil, albeit on a much faster timescale. However, the appearance of the 100 amu fragment ion in the single-photon VUV experiment presented in Section 2.2. shows that the multi-photon route is not an exclusive one, unlike what has been seen so far for the 84 amu fragment ion of uracil. Additionally, the multi-photon appearance of the 100 amu fragment ion from 2-TU does not seem to be as wavelength selective as what is observed for the 84 amu fragment ion of uracil [7–9]. In a mass spectrum presented by Yu *et al*., created though multi-photon probing using a 290 nm pump, the 100 amu fragment was still observed for 2-TU [10]. This excludes the idea that dynamics are being launched exclusively on states higher than the S_2.

However, it should be noted that in the mass spectra of 4-thiouracil and 2,4-dithiouracil by Hecht *et al*. both show peaks at 100 amu and 116 amu, respectively, which also indicate a loss of 28 amu from their respective parent structures [4]. As there is no oxygen atom on 2,4-dithiouracil, this may indicate that the 28 amu loss is instead something common to the three thionated uracil molecules (i.e. N_2 or HCNH) rather than CO. Unfortunately, the results presented here do not allow us to draw conclusion to one model or another. Despite these uncertainties, this discussion here may ultimately show that the loss of the 28 amu fragment may prove crucial in investigating the subtle differences in the dynamics between the thiouracils and uracil.

5. References

1. Jochims, H.W.; Schwell, M.; Baumgärtel, H.; Leach, S. Photoion mass spectrometry of adenine, thymine and uracil in the 6-22 eV photon energy range. *Chem. Phys.* **2005**, *314*, 263–282, doi:10.1016/j.chemphys.2005.03.008.

2. Denifl, S.; Sonnweber, B.; Hanel, G.; Scheier, P. Threshold electron impact ionization studies of uracil. *Int. J. ofMass Spectrom.* **2004**, *238*, 47–53, doi:10.1016/j.ijms.2004.07.010.

3. Coupier, B.; Farizon, B.; Farizon, M.; Gaillard, M.J.; Gobet, F.; De Castro Faria, N. V.; Jalbert, G.; Ouaskit, S.; Carré, M.; Gstir, B.; et al. Inelastic interactions of protons and electrons with biologically relevant molecules. *Eur. Phys. J. D* **2002**, *20*, 459–468, doi:10.1140/epjd/e2002-00166-3.

4. Hecht, S.M.; Gupta, A.S.; Leonard, N.J. Position of uridine thiation: The identification of minor nucleosides from transfer RNA by mass spectrometry. *BBA Sect. Nucleic Acids Protein Synth.* **1969**, *182*, 444–448, doi:10.1016/0005-2787(69)90195-6.

5. Rice, J.M.; Dudek, G.O.; Barber, M. Mass Spectra of Nucleic Acid Derivatives. Pyrimidines. *J. Am. Chem. Soc.* **1965**, *87*, 4569–4576, doi:10.1021/ja00948a029.

6. Nelson, C.C.; McCloskey, J.A. Collision-induced dissociation of uracil and its derivatives. *J. Am. Soc. Mass Spectrom.* **1994**, *5*, 339–349, doi:10.1016/1044-0305(94)85049-6.

7. Barc, B.; Ryszka, M.; Spurrell, J.; Dampc, M.; Limão-Vieira, P.; Parajuli, R.; Mason, N.J.; Eden, S. Multi-photon ionization and fragmentation of uracil: Neutral excited-state ring opening and hydration effects. *J. Chem. Phys.* **2013**, *139*, doi:10.1063/1.4851476.

8. Ghafur, O.; Crane, S.W.; Ryszka, M.; Bockova, J.; Rebelo, A.; Saalbach, L.; De Camillis, S.; Greenwood, J.B.; Eden, S.; Townsend, D. Ultraviolet relaxation dynamics in uracil: Time-resolved photoion yield studies using a laser-based thermal desorption source. *J. Chem. Phys.* **2018**, *149*, 034301, doi:10.1063/1.5034419.

9. Ryszka, M.; Pandey, R.; Rizk, C.; Tabet, J.; Barc, B.; Dampc, M.; Mason, N.J.; Eden, S. Dissociative multi-photon ionization of isolated uracil and uracil-adenine complexes. *Int. J. Mass Spectrom.* **2016**, *396*, 48–54, doi:10.1016/j.ijms.2015.12.006.

10. Yu, H.; Sanchez-Rodriguez, J.A.; Pollum, M.; Crespo-Hernández, C.E.; Mai, S.; Marquetand, P.; González, L.; Ullrich, S. Internal conversion and intersystem crossing pathways in UV excited, isolated uracils and their implications in prebiotic chemistry. *Phys. Chem. Chem. Phys.* **2016**, *18*, 20168–20176, doi:10.1039/c6cp01790h.
